# Inpatient pediatric antimicrobial use for respiratory infections during the RSV surge

**DOI:** 10.1017/ash.2023.213

**Published:** 2023-09-29

**Authors:** Aaron Hunt, Rodrigo Burgos, Alfredo Mena Lora

## Abstract

**Background:** In the United States, pneumonia causes >100,000 pediatric hospitalizations annually. On November 4, 2022, the CDC issued a Heath Advisory concerning an upcoming surge of respiratory illnesses including SARS-CoV-2, influenza, and respiratory syncytial virus (RSV). Differentiating between viral and bacterial causes is challenging and can lead to antimicrobial overuse. Currently, tools are being developed to distinguish between viral and bacterial pneumonia. The VALS-DANCE Pneumonia Etiology Predictor (PEP) provides clinical scoring criteria (Fig. 1) to determine probable cause of pneumonia with 93.1% sensitivity for bacterial pneumonia. Scores >11 have a >25% likelihood of having bacterial etiology. Given that antimicrobial exposure increases resistance rates, disrupts natural flora, and increases the risk of side effects, a core goal of researchers is to develop ways to promote stewardship and reduce inappropriate use. We assessed the patterns of use for antimicrobials in pediatric patients admitted with pneumonia at our institution. **Methods:** This retrospective review included pediatric cases admitted to an urban safety-net community hospital from July 22, 2022, to December 16, 2022. A daily list of all patients receiving antimicrobials was reviewed, and pediatric patients with diagnosis of a respiratory infection were included. Patients with additional indications for antimicrobial therapy, diagnosis of bronchitis, incomplete records, or without complete information were excluded from the scoring criteria. The primary objective was to assess the appropriateness of antimicrobial use for pneumonia, defined as use consistent with PEP scoring recommendations. **Results:** Of 53 patients reviewed, 37 met inclusion criteria. Of 37 patients, 22 (59.5%) met study criteria for appropriate therapy. The 15 patients (40.5%) who were inappropriate for treatment received an average of 4.67 ± 1.91 days of antibiotics. Of these 15 patients, 11 (73.3%) also had a positive viral test, further increasing the likelihood of a viral etiology. This subgroup had an average antibiotic exposure of 4.27 ± 1.79 days. Documented rationale for therapy included severity of illness (4 of 11), radiograph consolidation (4 of 11), and provider disagreement with radiograph interpretation (3 of 11). **Conclusions:** Pediatric respiratory infections represent a significant opportunity for antimicrobial stewardship. In this study, as many as 40% of pediatric patients may have received unnecessary antibiotic exposure. Use of the VALS-DANCE criteria may help clinicians identify patients with low likelihood of bacterial infection and reduce antimicrobial use. The national surge of viral infections serves to highlight the vital importance of appropriate diagnostic stewardship.

**Figure:**
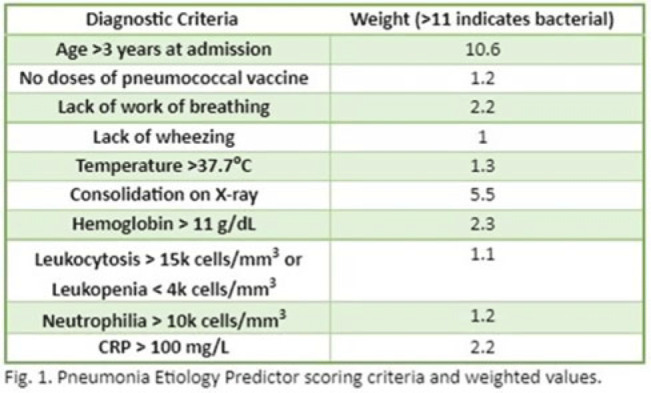

**Figure:**
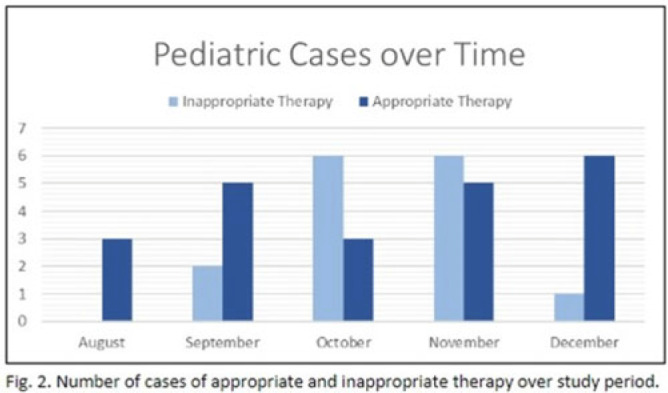

**Figure:**
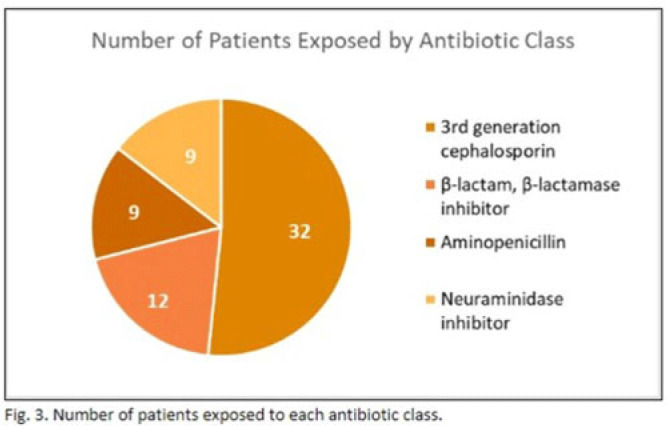

**Disclosure:** None

